# Effect of subthalamic coordinated reset deep brain stimulation on Parkinsonian gait

**DOI:** 10.3389/fninf.2023.1185723

**Published:** 2023-08-24

**Authors:** Kai M. Bosley, Ziling Luo, Sana Amoozegar, Kit Acedillo, Kanon Nakajima, Luke A. Johnson, Jerrold L. Vitek, Jing Wang

**Affiliations:** ^1^Department of Neurology, University of Minnesota, Minneapolis, MN, United States; ^2^Neuroscience Program, Macalester College, Saint Paul, MN, United States

**Keywords:** Parkinson's disease, subthalamic nucleus, coordinated reset, gait, non-human primate

## Abstract

**Introduction:**

Coordinated Reset Deep Brain Stimulation (CR DBS) is a novel DBS approach for treating Parkinson's disease (PD) that uses lower levels of burst stimulation through multiple contacts of the DBS lead. Though CR DBS has been demonstrated to have sustained therapeutic effects on rigidity, tremor, bradykinesia, and akinesia following cessation of stimulation, i.e., carryover effect, its effect on Parkinsonian gait has not been well studied. Impaired gait is a disabling symptom of PD, often associated with a higher risk of falling and a reduced quality of life. The goal of this study was to explore the carryover effect of subthalamic CR DBS on Parkinsonian gait.

**Methods:**

Three non-human primates (NHPs) were rendered Parkinsonian and implanted with a DBS lead in the subthalamic nucleus (STN). For each animal, STN CR DBS was delivered for several hours per day across five consecutive days. A clinical rating scale modified for NHP use (mUPDRS) was administered every morning to monitor the carryover effect of CR DBS on rigidity, tremor, akinesia, and bradykinesia. Gait was assessed quantitatively before and after STN CR DBS. The stride length and swing speed were calculated and compared to the baseline, pre-stimulation condition.

**Results:**

In all three animals, carryover improvements in rigidity, bradykinesia, and akinesia were observed after CR DBS. Increased swing speed was observed in all the animals; however, improvement in stride length was only observed in NHP B2. In addition, STN CR DBS using two different burst frequencies was evaluated in NHP B2, and differential effects on the mUPDRS score and gait were observed.

**Discussion:**

Although preliminary, our results indicate that STN CR DBS can improve Parkinsonian gait together with other motor signs when stimulation parameters are properly selected. This study further supports the continued development of CR DBS as a novel therapy for PD and highlights the importance of parameter selection in its clinical application.

## Introduction

Parkinson's disease (PD) is a progressive neurodegenerative disorder characterized by tremors, akinesia, bradykinesia, rigidity, and impairment in gait and posture. Deep brain stimulation (DBS) is an effective treatment for advanced PD; however, it has been associated with side effects likely caused by the current spreading into unintended brain regions (Saint-Cyr et al., 2000; Deuschl et al., 2006; van Nuenen et al., 2008; Odekerken et al., 2013). Coordinated Reset DBS (CR DBS) is an innovative approach to DBS that uses lower levels of burst stimulation over multiple contacts of the DBS lead and was designed to desynchronize abnormal neuronal population synchrony (Tass, 2003). It has been demonstrated in preclinical and clinical studies that CR DBS in the subthalamic nucleus (STN) can induce therapeutic improvements on rigidity, tremor, akinesia, and bradykinesia that can be sustained even after stimulation cessation, i.e., carryover effect (Tass et al., 2012; Adamchic et al., 2014; Wang et al., 2016). However, the impact of CR DBS on Parkinsonian gait has not been explored. Gait impairment is a profoundly disabling symptom of PD, often associated with higher risks of falling and reduced quality of life (Gray and Hildebrand, 2000; Kelly et al., 2012). Gait disturbances in PD include shuffling gait, decreased amplitude of motion at the joints, reduced movement velocity, and shortened stride length (Svehlík et al., 2009). It has been demonstrated that Parkinsonian gait impairment is also associated with abnormal neuronal synchronization such as exaggerated beta oscillatory activity in the STN (Toledo et al., 2014; Anidi et al., 2018; Chen et al., 2019; Fim Neto et al., 2022), providing a strong rationale for applying CR DBS in order to desynchronize the neuronal activity associated with the impaired gait. We hypothesized that STN CR DBS will also produce carryover improvement on Parkinsonian gait in addition to rigidity, akinesia, and bradykinesia. In this study, we tested this hypothesis by investigating the carryover effect of STN CR DBS on a modified version of the Unified Parkinson's Disease Rating Scale (mUPDRS), as well as the stride length and swing speed during gait in the Parkinsonian non-human primate (NHP) model of PD. Stride length and swing speed (similar to gait speed) are two standard gait measures (Doyle et al., 2022) that have been used in numerous clinical studies for differentiating PD patients from healthy controls or evaluating the effect of therapeutic treatments on gait (Luo et al., 2015; di Biase et al., 2020; De Oliveira et al., 2021; Gandolfi et al., 2023; Johansson et al., 2023; Matsuno et al., 2023). In addition, we examined the relative effect of STN CR DBS using two different burst frequencies on Parkinsonian gait. This was motivated by computational modeling studies (Lysyansky et al., 2013; Manos and Zeitler, 2018; Manos et al., 2018) and our previous NHP study (Wang et al., 2022) showing that varying the CR DBS parameter settings, e.g., intensity, frequency, dosage, and shuffling time, can significantly alter the effect of CR DBS. A particular modeling study showed that changing the burst frequency of the stimulation pattern greatly impacted the desynchronizing effect of CR stimulation (Manos and Zeitler, 2018). Taken altogether, our results suggest that STN CR DBS may produce carryover improvement in Parkinsonian gait when the stimulation parameters are properly selected.

## Materials and methods

Animal care complied with the National Institutes of Health Guide for the Care and Use of Laboratory Animals, and all procedures were performed under a protocol approved by the Institutional Animal Care and Use Committee of the University of Minnesota.

### Animals and surgical procedures

Three adult female rhesus monkeys (Macaca mulatta; Animal P, 6 kg; B1, 10.5 kg; B2, 8.2 kg) were used. Each animal was implanted with a head restraint system and a scaled-down version of the DBS lead (NuMed Inc.) targeting the STN (0.63 mm diameter, 0.5 mm contact height, and 0.5 mm space between contacts; total contact number: 4 for P, 8 for B1&B2) using techniques established in the laboratory (Wang et al., 2016, 2022). In brief, pre-operative high-resolution CT and MRI images were merged to identify the STN and make the surgical plan. A chamber and head restraint post were implanted during an aseptic surgery following which microelectrode recording and stimulation techniques were used to map the sensorimotor region and the borders of the STN. A final recording track was made to determine the lead placement depth after which a DBS lead was implanted. The cable of the DBS lead was then routed to a separate dry chamber which allows connection to a programmable pulse generator (Animal P: Abbott; Animal B1&B2: Boston Scientific) for the delivery of CR DBS.

Animals were rendered Parkinsonian through intracarotid and intramuscular injections of the neurotoxin 1-methyl-4-phenyl-1,2,3,6-tetrahydropyridine (MPTP). The severity of Parkinsonian motor signs on the side of the body contralateral to the site of DBS implantation was assessed using a version of the Unified Parkinson's Disease Rating Scale modified for NHP use (mUPDRS). On a 4-point scale (0–3; 0 = unimpaired), the mUPDRS was used to rate rigidity, bradykinesia, akinesia, and tremor for both the upper and lower limbs, as well as food retrieval for the upper limb only (maximum = 27 points). Prior to DBS testing, all animals reached a mild to moderate Parkinsonian state (Yu et al., 2021), demonstrating mainly rigidity, akinesia, and bradykinesia [mUPDRS: 17.3 ± 0.5 (mean ± SD) in NHP P, 7.6 ± 0.2 in NHP B1 and 10.4 ± 0.4 in NHP B2]. Animal B1 and B2 did not present tremors while animal P had mild tremors. These mUPDRS assessments were performed 12 times across 4 weeks in animal P, 10 times over 2 weeks in animal B1, and 14 times across 4 weeks in animal B2.

**Figure 1:**
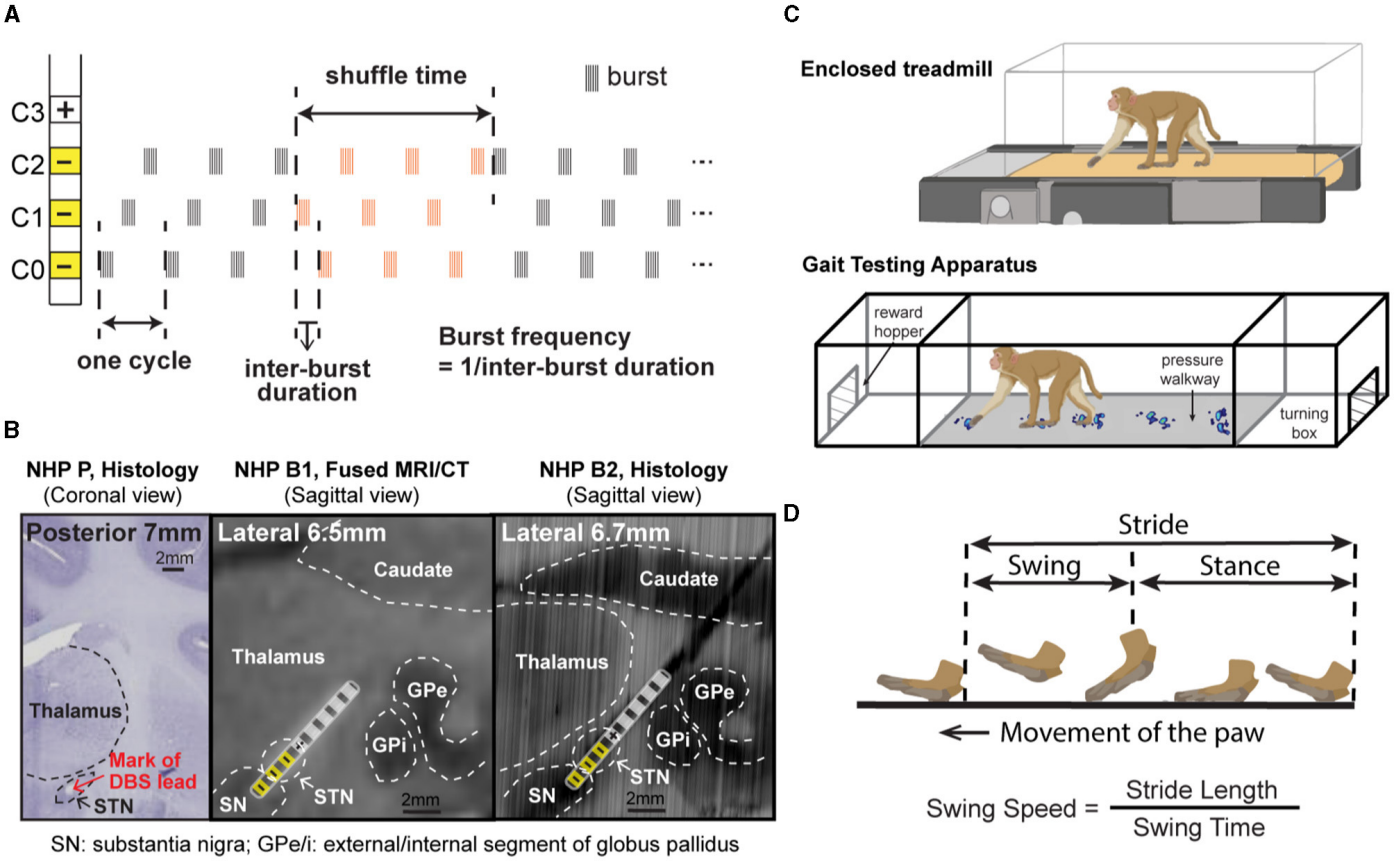
**(A)** CR DBS pattern showing the definition of shuffle time and burst frequency. **(B)** Histology or merged MRI/CT showing the location of the DBS lead in each animal. (Left) Coronal section from NHP P illustrating the relative location of the mark (red arrow) left by placement of the DBS lead in the STN. (Middle) Sagittal view of the merged MRI and CT images showing the estimated location of the DBS lead in NHP B1. (Right) Sagittal view of the DBS lead location reconstructed from the histology for NHP B2. Stimulation contacts used for CR DBS are indicated in yellow. **(C)** Demonstration of the treadmill system (top) used for NHP P and the Habit Trail system (bottom) used for NHP B1 and B2 for the assessment of gait. Both systems were enclosed using plexiglass. Modified from Doyle et al. (2022). **(D)** Definition of the swing and stance phases of gait and the calculation of swing speed.

At the end of the study, animals P and B2 were euthanized, and histology was performed. For NHP P, 50 μm coronal sections were stained with cresyl violet to identify the location of the DBS lead (Figure 1B left). For NHP B2, 40 μm coronal sections were imaged and visualized in a 3D slicer, with the sagittal view extracted to show the DBS lead location (Figure 1B right). Histology was not available for NHP B1, so a post-implantation CT was merged with the pre-operative MRI to verify the location of lead (Figure 1B middle).

### Experiment protocol

For each animal, CR DBS was delivered for 2 (NHP B1&B2) or 4 (NHP P) h per day for five consecutive days, followed by a period of post-treatment observation (at least 5 days) to characterize the carryover effects (post-CR days). The daily stimulation duration was chosen based on the time needed in each animal to observe therapeutic effects during CR DBS on a separate day before the study. The shortest time needed for the mUPDRS score to achieve its maximum reduction and plateau was chosen to be the daily stimulation duration. CR DBS parameters are demonstrated in Table 1. These parameters were determined based on previous studies (Tass et al., 2012; Wang et al., 2016, 2022) and the capability of the stimulators. CR DBS consisted of burst stimulation delivered through four most ventral contacts within the STN region (C0/C1/C2–, C3+) of the DBS lead (Figure 1A) in a pseudorandomized order. The stimulation intensity was determined as 1/3–1/2 of the intensity identified for the therapeutic traditional, isochronal DBS. The ON:OFF pattern and shuffle time used in NHP P were based on the previous studies (Tass et al., 2012; Wang et al., 2016), and those in NHP B1 and B2 were selected based on the device capability. A cycle is the time needed to deliver bursts through all selected cathodes, and the shuffle time is the time duration within which the stimulating contact order is kept the same before this order is pseudorandomly shuffled (Figure 1A). To assess the carryover effect of STN CR DBS on rigidity, bradykinesia, akinesia, and tremor, mUPDRS was performed prior to CR DBS on stimulation days and once daily in the morning on post-CR days. In NHP B2, two CR DBS settings were evaluated using the same experiment protocol, with burst frequency as the only different parameter (Table 1). In addition to the standard burst frequency (21 Hz) used in previous studies (Tass et al., 2012; Wang et al., 2016, 2022), a 27 Hz burst frequency was also evaluated. The 27 Hz burst frequency was chosen based on the oscillatory activity we observed in the local field potential signal detected in the STN that demonstrated a 27-Hz peak frequency. As modeling studies have suggested that CR DBS using a frequency at which the neuronal population is synchronized will be more effective (Tass, 2003), we hypothesized that CR DBS with a 27-Hz burst frequency will be more effective at improving all motor signs than that with a 21-Hz burst frequency in this animal. These two evaluation sessions were 10 months apart, and each session was initiated when a stable baseline mUPDRS score mentioned above was observed.

**Table 1:** Parameter settings for CR DBS.

**Parameters**	**NHP**		
	**P**	**B1**	**B2**
Intensity (mA)	0.2	0.1	0.16
Pulse width (μs)	125	120	120
Pulses/bursts	5	6	6
Intra-burst rate (Hz)	150	150	150
Burst frequency (Hz)	21	21	21, 27
On:off pattern (cycles)	3 On: 2 Off	NA	NA
Shuffle time (s)	0.143	10	10
Daily stim duration (hour)	4	2	2

**Figure 2:**
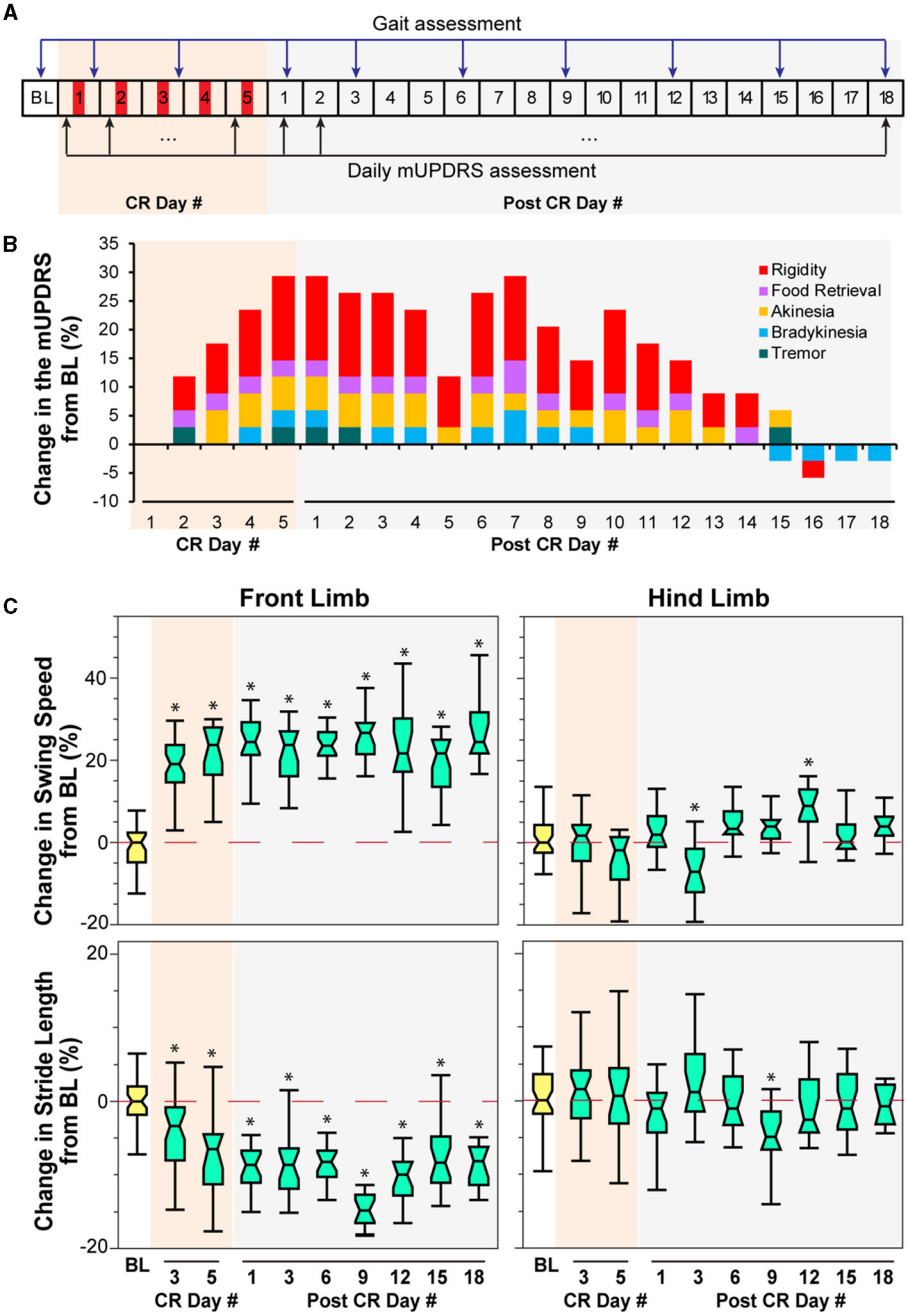
Effect of CR DBS on the mUPDRS and gait parameters in NHP P. **(A)** Schematic of the experiment protocol indicating the times when the mUPDRS and gait were assessed. Red panels: CR DBS, 4 h per Day. **(B)** Changes in the mUPDRS from baseline. The composite mUPDRS is further broken down to reveal the changes in individual subscores. **(C)** Changes in the swing speed and stride length at the front limb and hind limb from baseline. The ^*^symbol indicates a significant difference from BL (^*^P < 0.05).

**Figure 3:**
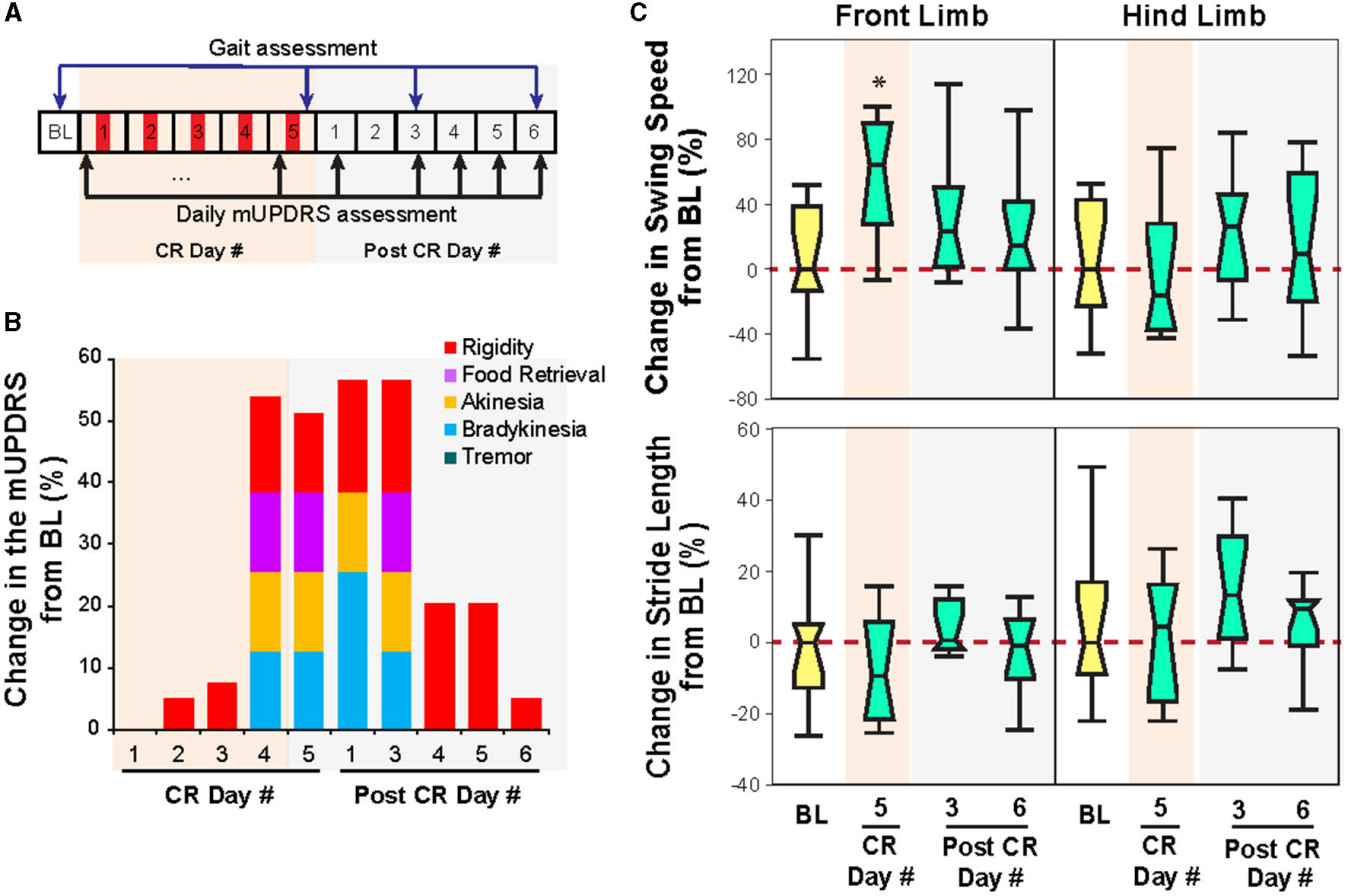
Effect of CR DBS on the mUPDRS and gait parameters in NHP B1. **(A)** Schematic of the experiment protocol. Red panels: CR DBS, 2 h per Day. **(B)** Changes in the mUPDRS and its subscores from baseline. **(C)** Changes in the swing speed and stride length at the front limb and hind limb from baseline. The ^*^symbol indicates a significant difference from BL (^*^P < 0.05).

**Figure 4:**
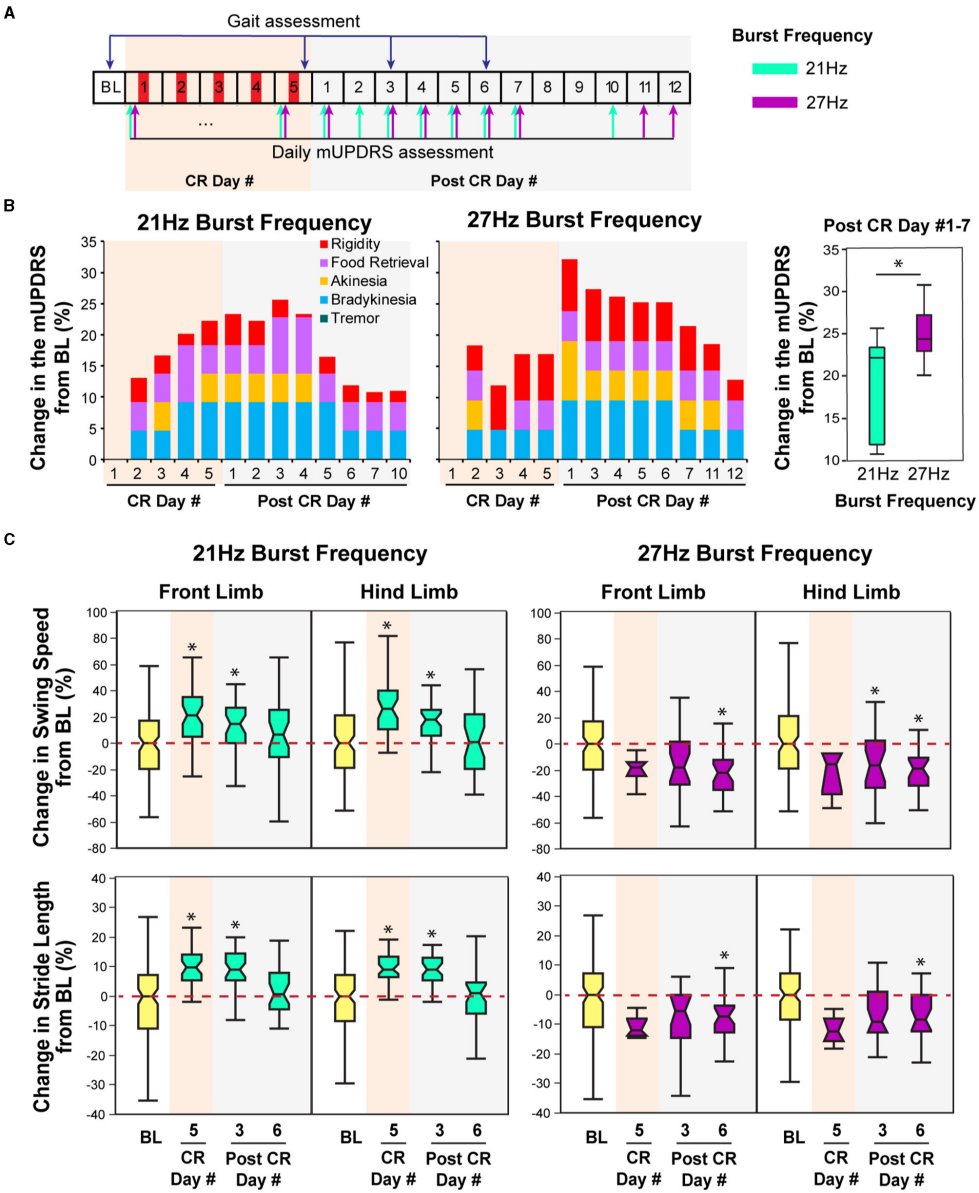
Effect of CR DBS using different burst frequencies on the mUPDRS and gait parameters in NHP B2. **(A)** Schematic of the experiment protocol. Red panels: CR DBS, 2 h per Day. The cyan and purple arrows indicate the mUPDRS assessment times for the CR DBS session using the 21 and 27 Hz burst frequencies, respectively. **(B)** Changes in the mUPDRS from baseline for the CR DBS session using 21 Hz (left) and 27Hz (middle) burst frequencies, as well as the comparison in the mUPDRS scores from post CR day #1–7 between these two sessions (right). **(C)** Changes in the swing speed and stride length at the front limb and hind limb from baseline after CR DBS using 21 Hz (left) and 27 Hz (right) burst frequencies. The ^*^symbol indicates a significant difference from BL (^*^P < 0.05).

The animal's gait was assessed using slightly different techniques due to a technical limitation at the time of the experiment. NHP P was ambulated in a treadmill system enclosed by plexiglass at a speed of 1.2 miles/hour (Figure 1C top). The movement of the animal's limbs was monitored using a motion capture system (Motion Analysis Corp.). NHPs B1 and B2 were ambulated in a gait testing apparatus (GTA) system in which natural, volitional gait data can be collected (Doyle et al., 2022). The GTA is an apparatus consisting of a plexiglass tunnel capped by two end enclosures, each of which is equipped with a hopper to deliver food or liquid reward (Figure 1C bottom). The animal's gait data were obtained using a pressure walkway mat (HR Walkway 4 VersaTek system, Tekscan, Inc.) in the tunnel. The GTA system allowed the NHP to walk naturally controlling its own pace, but the treadmill system required the NHP to walk continuously around the treadmill speed. CR DBS testing was not initiated until stable gait performance across days was observed. Prior to the DBS testing, animal P received 9 weeks of training on the treadmill, and animals B1 and B2 received 6 weeks and 4 weeks of training, respectively, in the GTA system. For all the NHPs, gait data were recorded before CR DBS, within 24 h after 5 days of CR DBS, and during the carryover period. The exact times of mUPDRS and gait assessments for each animal are shown in Figures 2A, 3A, 4A. Each gait evaluation session took ~20 min for animal P and ~30 min for animals B1 and B2. During the evaluation, animal P walked on the treadmill with brief breaks every 2 min, and animals B1 and B2 walked back and forward on the gait mat with brief breaks between passes.

### Data analysis

All mUPDRS scores obtained were converted to a percentage of change compared to the baseline score (the score obtained immediately prior to DBS on CR day 1): percentage change in mUPDRS score = 100^*^(baseline score – daily score)/baseline score. Positive changes indicate improvement in the mUPDRS scores. Given the potential natural fluctuation in the severity of Parkinsonian motor signs across days, we considered any changes smaller than 10% from the baseline score as fluctuations of the baseline level. The score was considered returned to baseline when the percentage change was reduced to 10%. The carryover effects, i.e., percent changes in the mUPDRS scores from the week immediately after 5 days of stimulation, of CR DBS using different burst frequencies in NHP B2 were compared with each other using the Wilcoxon test [χ^2^ (DoF, *N*)]. Using custom MATLAB functions, the movement trajectories at the front limb and hind limb from the motion analysis system and NHP steps collected from the pressure mat were analyzed, and gait parameters were then calculated. To evaluate changes in gait following CR DBS, we assessed the change in stride length and swing speed. As shown in Figure 1D, the distance that separated two consecutive points of initial contact of the same paw with the ground was referred to as the “stride length.” The amount of time that an animal spent with its paw in the air and away from the ground was considered “swing time.” “Swing speed” can be obtained by dividing “stride length” by “swing time.” The carryover effect of CR DBS on each gait parameter for the front and hind limbs on the left side (the side contralateral to the site of DBS implantation) was compared to the baseline using the Wilcoxon test followed by Steel's test with control = baseline. JMP (SAS Institute Inc., North Carolina, United States) was used to conduct statistical analyses, and alpha was corrected for multiple comparisons using the Bonferroni method.

## Results

Improvement in gait reflected by increased swing speed was observed in all three NHPs when STN CR DBS was delivered using a burst frequency of 21 Hz. Given the difference in gait assessment methods and experiment protocols, changes in mUPDRS ratings and gait parameters following the CR DBS are demonstrated separately for each animal.

### Animal P

Carryover improvement was observed in both the mUPDRS scores and swing speed during treadmill walking following STN CR DBS. Carryover improvement was observed in the mUPDRS score including improvements in each motor subscore (Figure 2B) compared to its baseline subscore (rigidity 3.5; food retrieval 2; akinesia 4; bradykinesia 3.5; tremor 4). Changes in the mUPDRS score showed gradual improvement during 5 days of STN CR DBS, reaching an improvement of 29.4% from the baseline on CR day 5. Following 5 days of CR DBS, the improvement in the mUPDRS was sustained for 12 days. The improvement in rigidity accounted for half or more of the total motor improvement. In addition to improvement in the mUPDRS score, we observed (Figure 2C) a 20% increase in swing speed noted at the NHP's front limb after stimulation on the 3rd and 5th CR days as well as on all the post-CR days [Figure 2C top, left, Wilcoxon test $\chi_{\left(9,231\right)}^{2}$ = 108.6, *p* < 0.0001 followed by Steel's test with control = BL, *p* < 0.05]. Opposite changes were observed in stride length at the same joint indicated by ~10% of decrease in most post-CR days [Figure 2C bottom, left, Wilcoxon test $\chi_{\left(9,231\right)}^{2}$ = 104.6, *p* < 0.0001 followed by Steel's test with control = BL, *p* < 0.05]. Limited changes in both parameters were observed at the hind limb, only showing significant changes in swing speed on post-CR days 3 and 12 [Wilcoxon test $\chi_{\left(9,231\right)}^{2}$ = 51.6, *p* < 0.0001 followed by Steel's test with control = BL, *p* < 0.05] and in stride length on post-CR day 9 [Wilcoxon test $\chi_{\left(9,231\right)}^{2}$ = 29.4, *p* = 0.0005 followed by Steel's test with control = BL, *p* < 0.05; Figure 2C right). Although the swing speed and stride length at the front limb did not return to the baseline level, their changes on post-CR days 12, 15, and 18 showed a trend of returning to the baseline.

### Animal B1

Similar to that observed in animal P, STN CR DBS was associated with carryover improvements in both the mUPDRS and swing speed in animal B1 but with a shorter duration of carryover benefits. As shown in Figure 3B, significant improvement in the mUPDRS (>50%) was observed starting from CR day 4, while only limited improvement (< 10%) was seen in the first 3 days of CR DBS compared to the baseline subscores (rigidity 2.8; food retrieval 1; akinesia 2; bradykinesia 2; tremor 0). The improvement was observed in rigidity, food retrieval, akinesia, and bradykinesia but not tremors as this animal did not demonstrate tremors in its Parkinsonian state. This large improvement in the mUPDRS score reduced significantly starting from the fourth day after 5 days of CR DBS and returned to a level (5%) close to the baseline. During ambulation in the habit trail system, increased swing speed at the front limb immediately after 5 days of CR DBS [Wilcoxon test $\chi_{\left(3,38\right)}^{2}$ = 8, *p* < 0.05 followed by Steel's test with control = BL, *p* < 0.05] was observed, while the swing speed at the hind limb and stride length did not change (Figure 3C). Consistent with the trend of change in the mUPDRS score after post-CR day 3, improvement in swing speed was only observed immediately after 5 days of CR DBS and diminished on post-CR day 3.

### Animal B2

In this animal, carryover improvement in the mUPDRS score was observed in both CR sessions using 21 and 27 Hz burst frequencies; however, improvement in gait parameters was only observed after CR DBS using the 21 Hz burst frequency (Figures 4B, C). The baseline mUPDRS subscores obtained prior to the 21 Hz session were 2.425 (rigidity), 2 (food retrieval), 3 (akinesia), 3.5 (bradykinesia), and 0 (tremor), and those prior to the 27 Hz session were 3 (rigidity), 1.5 (food retrieval), 3 (akinesia), 3 (bradykinesia), and 0 (tremor). CR DBS using the 21 Hz burst frequency produced up to 25.6% of carryover improvement in the mUPDRS score relative to the baseline (Figure 4B left). This improvement reduced over time and dropped to a level of 10% on post-CR day 7. During the CR session using the 27 Hz burst frequency, up to 32% of carryover improvement in the mUPDRS score was observed (Figure 4B middle). This carryover improvement was not reduced to a level close to 10% until post-CR day 12, indicating longer carryover benefits compared to the CR session using 21Hz burst frequency. In both CR sessions, improvement was observed in all the motor subscores except for tremors. Investigating the changes in the mUPDRS in the week following 5 days of CR DBS, greater carryover improvement was observed with CR DBS using 27 Hz burst frequency [Figure 4B right, Wilcoxon test $\chi_{\left(1,13\right)}^{2}$ = 4.6, *p* = 0.03] mostly due to the greater improvement in rigidity (Figure 4B left and middle).

In contrast to the changes in mUPDRS, changes in gait parameters demonstrated carryover improvement after CR DBS using the 21 Hz burst frequency but not with the 27 Hz burst frequency. During the CR session using the 21 Hz burst frequency, increases in the swing speed and stride length relative to the baseline were observed at both the front limb and hind limb, immediately following 5 days of CR DBS and on post-CR day 3 (Figure 4C left). Both parameters returned to the baseline level on post-CR day 6. CR DBS using 27 Hz burst frequency rather than improving swing speed and stride length had the opposite effect leading to longer swing times and shorter stride lengths on post-CR day 6 (Figure 4C right). The results of the statistical analysis on the gait parameters for NHP B2 are shown in Table 2.

**Table 2:** Statistical analysis for the gait parameters for NHP B2.

		**Wilcoxon test**			***p*****-value of Steel's test with control** = **BL**		
		χ^2^	**DoF**, ***N***	***p*** **-value**	**CR day 5**	**Post-CR day 3**	**Post-CR day 6**
**21 Hz burst frequency**							
Swing speed	Front limb	24.1	3, 240	< 0.0001	< 0.0001	0.0151	0.5383
	Hind limb	34.8	3, 240	< 0.0001	< 0.0001	0.0054	0.9989
Stride length	Front limb	49.3	3, 240	< 0.0001	< 0.0001	< 0.0001	0.7049
	Hind limb	52.1	3, 240	< 0.0001	< 0.0001	< 0.0001	0.9983
**27 Hz burst frequency**							
Swing speed	Front limb	21.5	3, 184	< 0.0001	0.1202	0.1654	< 0.0001
	Hind limb	25.2	3, 184	< 0.0001	0.0824	0.0249	< 0.0001
Stride length	Front limb	13.8	3, 184	0.0032	0.1044	0.1009	0.0169
	Hind limb	14.3	3, 184	0.0026	0.0596	0.0564	0.0358

## Discussion

Previous studies have demonstrated the efficacy of STN CR DBS in improving Parkinsonian motor signs including rigidity, akinesia, bradykinesia, and tremor. This study demonstrates that STN CR DBS might also improve Parkinsonian gait. The differential effects of CR DBS using different burst frequencies on gait parameters observed in NHP B2 (Figure 4C) also indicate that the selection of CR parameters, e.g., burst frequency, can significantly impact the efficacy of CR DBS on gait. Moreover, the potential different impact of varying the burst frequency on Parkinsonian gait and other motor signs, i.e., one burst frequency might be more efficient at improving gait, while the other might produce greater improvement on other motor signs, indicating the importance of parameters selection for treating specific PD symptoms. The findings from our research not only support our hypothesis that CR DBS can improve Parkinsonian gait but also can demonstrate the importance of parameter selection for CR DBS in order to achieve specific motor benefits.

### STN CR DBS can improve Parkinsonian gait

CR stimulation was developed through computational modeling studies performed by Peter Tass' group (Tass, 2003; Tass and Majtanik, 2006; Hauptmann et al., 2007; Lysyansky et al., 2011). This stimulation approach was designed to desynchronize the neuronal population by stimulating the neuronal subpopulations with a small amount of current in a phase-shifted manner, with the stimulation frequency determined as the frequency at which neurons were synchronized. As abnormal neuronal synchronization in the basal ganglia-thalamocortical network has been associated with the development of PD motor symptoms (Connolly et al., 2015; Neumann et al., 2017; Sanabria et al., 2017; Tinkhauser et al., 2018; Lofredi et al., 2019), pilot preclinical and clinical studies were conducted to explore the effect of CR DBS on PD motor signs (Tass et al., 2012; Adamchic et al., 2014; Wang et al., 2016). These studies demonstrated the acute and carryover therapeutic effects of STN CR DBS stimulated on a wide range of Parkinsonian motor signs, but its effect on Parkinsonian gait was not examined. This study fills the gap by demonstrating the potential therapeutic carryover improvement on Parkinsonian gait of the STN CR DBS using the 21 Hz burst frequency, indicated by the improved gait speed of the front limb in all animals (Figures 2C, 3C, 4C). Stride length was also improved in NHP B2 but reduced in NHP P after CR DBS. The reduced stride length in NHP P was likely due to the different gait assessment approach. NHP P was ambulated in an enclosed treadmill system with limited space. With increased gait speed that exceeded the treadmill speed, the animal reached the front of the treadmill which prevented further movement, resulting in reduced stride length. On the other hand, improvements in both the swing speed and stride length were observed in NHP B2 when the animal was ambulating naturally in the habit trail system although stride length was not improved in NHP B1. This finding indicates the importance of a gait assessment system that can quantitatively evaluate the naturalistic, volitional gait patterns (Doyle et al., 2022).

Exploring the effect of CR DBS on Parkinsonian gait is also a critical step toward the clinical translation of this novel DBS approach. Long-term follow-up studies have shown that certain aspects of gait function improve initially with traditional high-frequency DBS but then progressively worsen resulting in more pronounced asymmetry and dyscoordination (Krack et al., 2003; Volkmann et al., 2004; van Nuenen et al., 2008; Ravi et al., 2021). Although the results are preliminary, this study supports the hypothesis that STN CR DBS can improve Parkinsonian gait while using lower stimulation intensity than traditional DBS, in addition to the benefits CR DBS induced in rigidity, akinesia, bradykinesia, and tremor. Further research on more subjects to evaluate the longer-term therapeutic effects of CR DBS will be needed to confirm our findings. Additional studies (Conway et al., 2021; Seger et al., 2021; Su et al., 2022; Cavallieri et al., 2023; Pourahmad et al., 2023) to compare the effect of CR DBS on gait with that of traditional DBS will also be required for the clinical translation of CR DBS.

### Differential effects of different CR burst frequencies on gait and other motor signs

Computational modeling studies have shown that varying CR parameters, e.g., stimulation intensity, burst frequency, stimulation dosage, and number of stimulation contacts, can significantly impact the desynchronizing effect of CR stimulation (Lysyansky et al., 2013; Manos and Zeitler, 2018; Manos et al., 2018). Our previous study also demonstrated significantly greater carryover benefits associated with shuffled CR DBS compared to the non-shuffled pattern (Wang et al., 2022). Although the results are preliminary, this study is the first to show the differential effects on gait and other motor signs (rigidity, bradykinesia, and akinesia) after CR DBS using different burst frequencies, with one parameter (burst frequency 21 Hz) producing greater carryover benefits on gait, while the other (27 Hz) produced greater benefits on other motor signs (Figure 4C). This might indicate that a specific burst frequency is required to optimize the effect of CR DBS on Parkinsonian gait, while a different burst frequency is required for achieving optimal improvement on rigidity, akinesia, and bradykinesia. As only two burst frequencies were evaluated in animal B2, it is also possible that the optimal burst frequency that can improve both gait and other motor signs has not been identified. Additional explorations in the effect of CR DBS using a wider range of burst frequencies will be needed to further investigate the impact of burst frequency on different Parkinsonian motor signs and even non-motor signs.

### Limitations and future directions

Even though all three animals showed significant improvement in gait speed after CR DBS, there were some limitations within this study. The effects of STN CR DBS with different burst frequencies were not investigated in animals P and B1. This was attributable to the length of time necessary to examine each CR DBS setting and the limited time for evaluating CR DBS in these two animals. Due to the various capabilities of the device available at the time of the experiments and the varying lengths of daily stimulation necessary to produce a sustained therapeutic effect, animals P and B1/B2 were subjected to different ON:OFF patterns, shuffling times, and stimulation durations, while other CR parameters were the same. Different gait assessment systems, i.e., treadmill and habit trail systems, were used, which resulted in different observations of the change in stride length. As discussed above, the assessment of the natural gait in the habit trail system is superior to that of the passive gait movement in the treadmill, making it critical for future explorations into the effects of CR DBS (Doyle et al., 2022). Another limitation of this study is that carryover assessment was terminated when the mUPDRS score returned to the baseline and an offline gait data analysis was performed afterward to investigate the effect of CR DBS on gait. Therefore, we were not able to observe the returning of gait parameters to the baseline in animal P and the 27 Hz CR DBS session in animal B2. Future studies are needed to systematically evaluate the impact of different CR parameters on Parkinsonian gait.

Despite these limitations, this study provides valuable insight into the effect of STN CR DBS on Parkinsonian gait and the potential impact of varying CR parameters on gait and other motor signs. These findings support the development of CR DBS as a novel DBS strategy that can be customized for each patient and further advance the translation of this novel therapy into clinical application.

## Data availability statement

The raw data supporting the conclusions of this article will be made available by the authors, without undue reservation.

## Ethics statement

The animal study was reviewed and approved by The Institutional Animal Care and Use Committee.

## Author contributions

JW conceived and designed the experiments. LJ, JW, KB, and ZL contributed to animal instrumentation. JW, KB, ZL, and KA acquired the data. KB, ZL, and KN analyzed the data. KB, SA, JW, LJ, and JV were involved in drafting the article. All authors have reviewed the article and approved the final version for submission.
